# Long-Term Clinical Outcomes After Procedural Hemopericardium Requiring Pericardiocentesis During Atrial Fibrillation Ablation

**DOI:** 10.3390/jcdd13090407

**Published:** 2026-08-24

**Authors:** Soohyun Kim, Soyoon Park, Hwajung Kim, Young Choi, Yong-Seog Oh, Sung-Hwan Kim

**Affiliations:** 1 Division of Cardiology, Department of Internal Medicine, Incheon St. Mary’s Hospital, College of Medicine, The Catholic University of Korea, Incheon 21431, Republic of Korea; kimsoohyun@catholic.ac.kr; 2 Catholic Research Institute for Intractable Cardiovascular Disease, College of Medicine, The Catholic University of Korea, Seoul 06591, Republic of Korea; syoon007@catholic.ac.kr (S.P.); hjkim@catholic.ac.kr (H.K.); superstar@catholic.ac.kr (Y.C.); oys@catholic.ac.kr (Y.-S.O.); 3 Division of Cardiology, Department of Internal Medicine, Seoul St. Mary’s Hospital, College of Medicine, The Catholic University of Korea, Seoul 06591, Republic of Korea; 4 Division of Cardiology, Department of Internal Medicine, Yeouido St. Mary’s Hospital, College of Medicine, The Catholic University of Korea, Seoul 07345, Republic of Korea; 5 Division of Cardiology, Asan Medical Center, College of Medicine, University of Ulsan, Olympic-ro 43-gil, Songpa-gu, Seoul 05505, Republic of Korea

**Keywords:** atrial fibrillation, catheter ablation, cardiac tamponade, pericardial effusion, colchicine

## Abstract

Hemopericardium is a rare but serious complication of radiofrequency catheter ablation (RFCA) for atrial fibrillation (AF). While prompt pericardial drainage is effective, the long-term clinical course and the role of adjunctive anti-inflammatory therapy remain unclear. In this prospective single-center cohort study, we included patients who developed hemopericardium requiring pericardiocentesis during RFCA for AF. All patients underwent immediate percutaneous drainage. Colchicine (0.6 mg twice daily for 14 days) was prescribed at the operator’s discretion. The primary outcome was freedom from AF or atrial tachycardia (AT) at 12 months after a 3-month blanking period. Among 2133 patients undergoing RFCA, 75 (3.5%) developed hemopericardium. Of these, 21 received colchicine and 54 received usual care. During a median follow-up of 367 days, freedom from AF/AT did not differ between groups (76.2% vs. 74.1%, log-rank *p* = 0.93). Residual pericardial effusion was infrequent and resolved in all patients by 3 months. Although colchicine was not associated with a reduction in atrial arrhythmia recurrence, patients receiving colchicine had lower CRP levels at 3 months than those receiving usual care (*p* = 0.021). However, treatment-limiting adverse events occurred in 6 of 21 patients (28.6%) receiving colchicine. No cases of constrictive pericarditis were observed. In patients with procedural hemopericardium during AF ablation, prompt pericardial drainage was associated with favorable clinical outcomes and absence of long-term pericardial sequelae. Adjunctive colchicine therapy was not associated with a significant reduction in arrhythmia recurrence; however, given the small sample size and limited number of events, a clinically meaningful treatment effect cannot be excluded.

## 1. Introduction

Atrial fibrillation (AF) is a common sustained tachyarrhythmia, with a lifetime risk of approximately one in three individuals [1]. Radiofrequency catheter ablation (RFCA) has become an established rhythm-control strategy for symptomatic AF, offering effective symptom relief and improved quality of life. Despite advances in mapping systems, catheter technology, and procedural techniques, peri-procedural complications remain an important clinical concern.

Among these, cardiac tamponade is one of the most serious complications and remains a leading cause of peri-procedural mortality, with an estimated rate of 0.9–1.7% [2,3,4,5]. Cardiac tamponade typically results from mechanical perforation during transseptal puncture or catheter manipulation, or from excessive energy delivery during ablation [6]. In most cases, prompt percutaneous pericardial drainage is sufficient, although a minority of patients require surgical intervention [7]. Hemopericardium during AF ablation may provoke acute pericardial inflammation and has been hypothesized to contribute to adverse pericardial remodeling, including effusive-constrictive physiology [8]. However, data regarding the long-term clinical course of procedural hemopericardium remain limited. In particular, it is unclear whether hemopericardium influences subsequent atrial arrhythmia recurrence after ablation. Colchicine is an established anti-inflammatory drug for postoperative and recurrent pericarditis [9,10]; however, the efficacy of colchicine for recurrence of AF after RFCA remains controversial. Moreover, the role of colchicine in patients who develop hemopericardium during AF ablation has not been specifically evaluated. Therefore, this study aimed to evaluate the clinical course of patients who developed hemopericardium during RFCA for AF, with a focus on long-term rhythm outcomes and pericardial sequelae. In addition, we sought to assess whether adjunctive colchicine therapy provides incremental benefit in this clinical setting.

## 2. Materials and Methods

### 2.1. Study Design and Patient Selection

**Figure 1:**
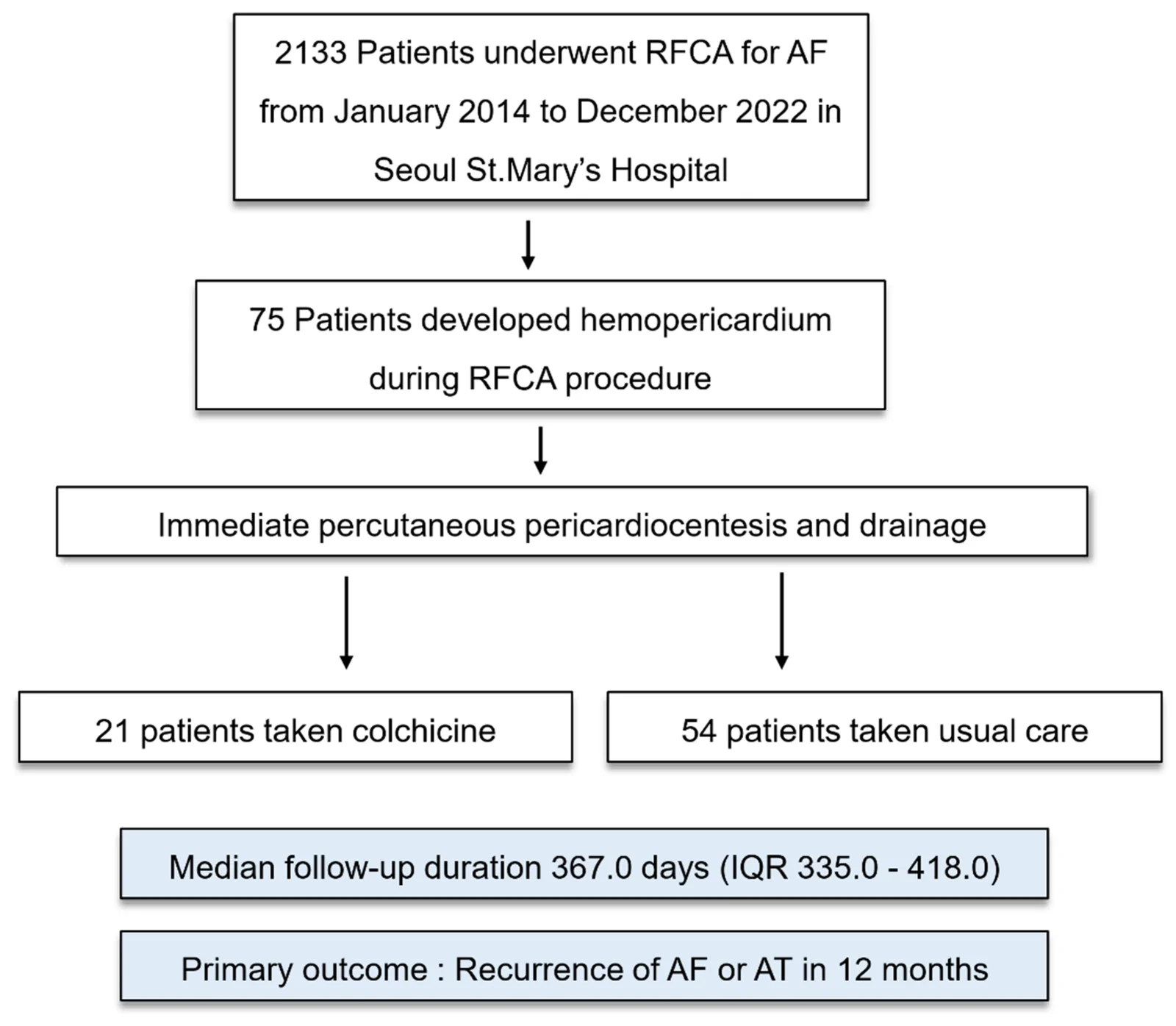
Study flow chart.

This was a prospective, single-center observational cohort study conducted at Seoul St. Mary’s Hospital, Republic of Korea, between January 2014 and December 2022. During the study period, 2133 patients underwent RFCA for AF. The present analysis included patients who developed intraprocedural hemopericardium requiring pericardiocentesis during RFCA. Patients with delayed hemopericardium occurring after completion of the procedure were not included. A total of 75 patients (3.5%) met the eligibility criteria and constituted the study cohort (Figure 1). Among these patients, 21 received colchicine beginning on post-ablation day 1 (colchicine group), whereas 54 received usual care without colchicine (usual care group). Colchicine was administered at a dose of 0.6 mg twice daily for 14 days. Treatment allocation was not randomized or based on predefined eligibility criteria but was determined at the treating operator’s discretion. Therefore, potential treatment selection bias and confounding by indication were considered in the interpretation of between-group comparisons. The Institutional Review Board of Seoul St. Mary’s Hospital approved the study protocol (KC13EISI0678; 8 May 2014), and all patients provided written informed consent. The study was conducted in accordance with the Declaration of Helsinki.

### 2.2. Procedure and Periprocedural Management

All patients underwent RFCA with moderate-to-deep sedation with non-invasive ventilation and administration of analgesics and sedatives. The procedure was guided using a CARTO 3 (Biosense Webster, Diamond Bar, CA, USA) or Ensite Navx ^TM^ (Abbott, St. Paul, MN, USA) three-dimensional electroanatomic mapping system. The ablation procedure was performed using radiofrequency energy with an open irrigated catheter (Coolflex, Abbott, St. Paul, MN, USA; or Thermocool, Biosense Webster, Diamond Bar, CA, USA). Oral anticoagulant was interrupted 1 day before the procedure, and unfractionated heparin was administered intravenously to target an activated clotting time >300 s during the procedure. For the lesion set, bilateral circumferential pulmonary vein (PV) antrum ablation was performed with radiofrequency energy of 25–35 W for 20–30 s, and the need for additional lines was left to the operators’ discretion. Redo ablation procedures included treatment of a conduction gap within previously isolated pulmonary veins and clinical tachycardia if warranted. If cardiac tamponade or hemopericardium was suspected, immediate transthoracic echocardiography or intracardiac echocardiography was performed. Procedural hemopericardium was defined as intraprocedural pericardial blood accumulation requiring urgent pericardiocentesis based on echocardiographic findings and clinical judgment. The cohort therefore included patients with hemodynamically significant cardiac tamponade as well as those with clinically significant or rapidly accumulating hemopericardium requiring drainage before overt hemodynamic collapse. Percutaneous pericardiocentesis was performed promptly under fluoroscopic guidance when clinically significant hemopericardium requiring drainage was identified. Auto-transfusion of drained blood was performed using the previously placed transfemoral catheter if possible, followed by natural drainage for less than 24 h. Continuation of ablation after pericardiocentesis was based on operators’ discretion. Patients visited the outpatient clinic at 1, 3, 6, and 12 months after RFCA. Arrhythmia recurrence after the 3-month blanking period was defined as documented AF or atrial tachycardia (AT) on a 12-lead electrocardiogram (ECG) or lasting at least 30 s on Holter monitoring. Scheduled ECGs were obtained during outpatient follow-up, and 24 h Holter monitoring was performed at 6 and 12 months. Additional ECG or ambulatory monitoring was performed in patients reporting symptoms suggestive of recurrence. The primary endpoint was evaluated irrespective of concomitant antiarrhythmic drug use. Transthoracic echocardiography was performed at the 3-month visit. Long-term oral anticoagulants were continued after the procedure based on CHA_2_DS_2_-VASc score, and antiarrhythmic drugs were restarted in those patients at physicians’ discretion. 

### 2.3. Endpoints

The primary endpoint was freedom from any atrial arrhythmia (AF or AT) at 12 months. The secondary endpoints included incidence of residual pericardial effusion, recurrent pericardial effusion, and development of constrictive pericarditis as assessed by transthoracic echocardiography during follow-up.

### 2.4. Statistical Analysis

Categorical data are presented as frequency and proportion and were compared with the chi-square (χ^2^) test (or Fisher’s exact test when any expected count was <5 for a 2 × 2 table). Continuous variables are expressed as mean ± standard deviation or median and interquartile range (IQR) based on normality and were compared using Student’s *t*-test or Mann–Whitney U test. Survival curves were constructed using the Kaplan–Meier method and were compared using the log-rank test. Independent predictors were identified with multivariable Cox proportional hazards model and expressed as hazard ratio (HR) with 95% confidence interval (CI). Adjustment was made for age, sex, diabetes mellitus, hypertension, paroxysmal versus persistent AF, duration of AF, heart failure, left ventricular ejection fraction, and RF ablation for extra-PV substrates. Given the limited number of recurrence events relative to the number of covariates, the multivariable analysis was considered exploratory, and adjusted estimates were interpreted cautiously. No formal sample-size calculation was performed because the study cohort comprised all eligible patients who developed intraprocedural hemopericardium requiring pericardiocentesis during the predefined study period. All statistical tests were two-sided, and a *p* value < 0.05 was considered statistically significant. Statistical analyses were performed using R version 4.5.1 (R Foundation for Statistical Computing, Vienna, Austria).

## 3. Results

### 3.1. Baseline and Procedural Characteristics

Among the 75 enrolled patients, the mean age was 65.7 ± 11.1 years, and 24 (32.0%) were female. Baseline characteristics are summarized in Table 1. Ablation of extra-PV substrate was performed in 43 (57.3%) patients. The median total procedure time was 219.0 min (IQR 185.0–250.5 min), and the median time from initiation of the procedure to drainage of hemopericardium was 158.5 min (IQR 124.0–200.0 min). Of 75 hemopericardium events observed during the procedure, 32 (42.7%) occurred during PV ablation, 7 (9.3%) during roof line, 6 (8.0%) during perimitral isthmus, 4 (5.3%) during right atrium, 1 (1.3%) during anterior wall and 4 (5.3%) during initial electro-anatomical mapping of the left atrium (Supplementary Table S1). The RFCA procedure was continued after immediate pericardiocentesis and completed as planned in 46 of 75 patients with hemopericardium (10 [47.6%] in colchicine group vs. 36 [66.7%] in usual care group, *p* = 0.21). The procedural characteristics are summarized in Table 2. All patients, including subjects who developed hemopericardium during mapping of the left atrium, received RFCA for PV isolation. Auto-transfusion of drained blood was performed in 57 (76.0%) patients and 43 (57.3%) received conventional transfusion with packed red blood cells (RBCs). All drainage catheters were removed within 24 h. Four patients required surgical management for persistent bleeding. After the RFCA procedure, intravenous protamine was administered to 61 (81.3%) patients, and all patients received an intravenous corticosteroid (methylprednisolone 0.5 mg/kg) immediately and on the day after RFCA. Oral anticoagulant was not resumed on the day after the procedure in 48 patients (64%). The duration of anticoagulant interruption was shorter in the colchicine group than in the usual care group (median 0.0 [IQR 0.0–1.0] vs. 3.0 [IQR 0.0–7.0] days, *p* = 0.002). The median hospital stay was 4.0 days (IQR 3.0–5.0 days). Laboratory tests including hemoglobin and C-reactive protein (CRP) levels were analyzed. Baseline hemoglobin and post-procedural hemoglobin levels were higher in the colchicine group, whereas the magnitude of hemoglobin decline did not differ significantly between groups. Postoperative day 1 CRP levels did not differ significantly between groups. In an exploratory analysis, CRP levels measured at 3 months were significantly lower in the colchicine group than in the usual-care group (median 0.025 [IQR 0.010–0.050] vs. 0.050 [IQR 0.030–0.163] mg/dL, *p* = 0.021). Adverse events were not prospectively collected using a standardized surveillance protocol. However, clinically documented adverse reactions resulting in premature colchicine discontinuation were identified from the medical records. Treatment-limiting adverse events occurred in six patients (28.6%) receiving colchicine, including gastrointestinal intolerance in five patients and dyspnea on exertion in one patient.

### 3.2. Study Endpoints

**Figure 2:**
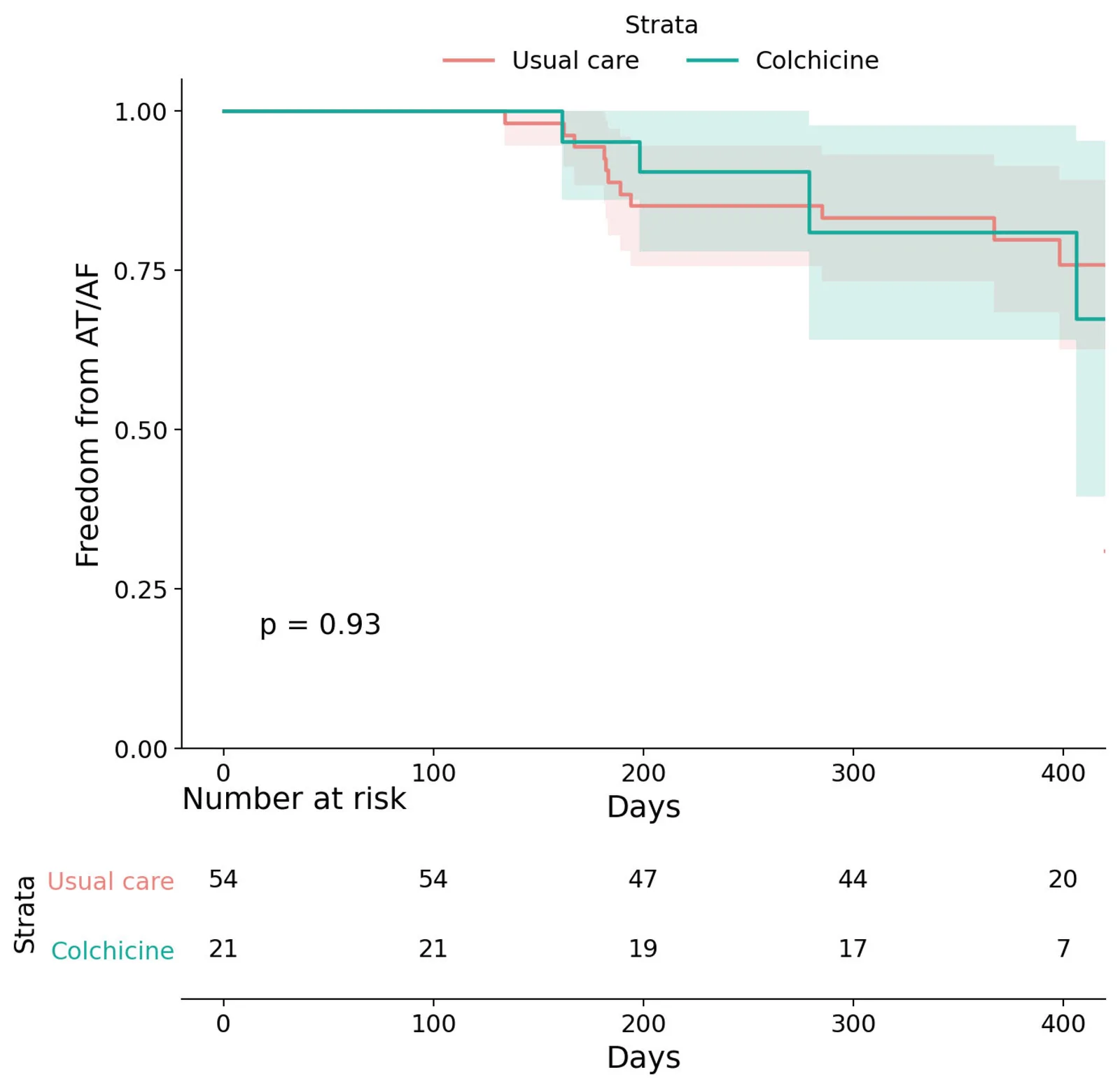
Kaplan–Meier estimates of freedom from AF or AT after the 3-month blanking period in patients who developed hemopericardium treated with pericardiocentesis, according to adjunctive colchicine therapy or usual care.

During a median follow-up of 367.0 days (IQR 335.0–418.0 days), freedom from atrial arrhythmia including AF and AT irrespective of antiarrhythmic drugs was similar in the groups (76.2% in the colchicine group and 74.1% in the usual care group, log-rank *p* = 0.93; Figure 2). In univariate analysis, colchicine was not associated with lower risk of AF or AT recurrence. In multivariable Cox regression analysis, only paroxysmal AF was associated with lower risk of AF/AT recurrence at 12 months (adjusted HR 0.22, 95% CI 0.06–0.77; Table 3). However, given the limited number of outcome events relative to the number of covariates, this finding should be interpreted cautiously. Continuation of RFCA after hemopericardium management was not associated with arrhythmia recurrence. Residual pericardial effusion at 1 month was observed in one patient in the colchicine group and two in the usual care group, and regressed by 3 months. No cases of recurrent pericardial effusion or constrictive pericarditis were identified during follow-up.

## 4. Discussion

In this prospective cohort of patients who developed hemopericardium during RFCA for AF, several clinically relevant findings emerged. First, most cases were successfully managed with prompt percutaneous pericardial drainage, and no patient developed constrictive pericarditis during follow-up. Second, long-term rhythm outcomes were generally favorable following prompt management of procedural hemopericardium. Third, adjunctive colchicine therapy was not associated with a reduction in atrial arrhythmia recurrence.

### 4.1. Clinical Course of Hemopericardium During AF Ablation

Pericardial blood accumulation has been proposed to promote atrial arrhythmogenesis through mechanical compression, local inflammatory activation, and oxidative stress, creating a proinflammatory environment adjacent to the atrial myocardium [11,12]. Previous surgical studies have demonstrated that postoperative pericardial effusion is associated with increased atrial arrhythmias, and that adequate posterior pericardial drainage can mitigate postoperative AF risk [13,14,15]. In contrast to postoperative effusions, all hemopericardium events in our study were recognized during the procedure and managed immediately in the electrophysiology laboratory. No patient exhibited persistent pericardial effusion at 3 months, and constrictive physiology was not observed. These findings suggest that prompt evacuation of pericardial blood may have mitigated sustained pericardial irritation and subsequent fibrotic remodeling, thereby potentially limiting long-term arrhythmogenic impact, even in patients in whom the ablation procedure was prematurely terminated.

### 4.2. Colchicine and Post-Ablation Arrhythmia Recurrence

Colchicine is an anti-inflammatory drug with established efficacy in acute and recurrent pericarditis and in post-cardiac injury syndromes [16,17,18]. Its mechanisms include inhibition of tubulin polymerization and suppression of inflammatory cell activation, resulting in attenuation of cytokine-mediated inflammatory cascades [19,20,21]. In surgical populations, prior studies have reported that colchicine reduced postoperative AF, particularly within the early postoperative period, likely mediated by attenuation of acute inflammatory responses [22]. However, data regarding its role in preventing AF recurrence after RFCA remain inconsistent, and the precise mechanistic pathways in the post-ablation setting are not fully elucidated.

Radiofrequency ablation induces myocardial injury characterized by coagulative necrosis, tissue edema, oxidative stress, and transient autonomic imbalance [23]. Early atrial tachyarrhythmia recurrence during the 3-month blanking period is thought to be largely driven by these transient inflammatory and healing processes, whereas late recurrence more commonly reflects pulmonary vein reconnection or progressive atrial substrate remodeling. This pathophysiologic distinction provides a rationale for testing anti-inflammatory therapies to reduce early recurrence.

Several randomized studies have demonstrated that colchicine may reduce early AF recurrence within the blanking period [24]. However, data beyond the blanking period have been inconsistent. Some studies have suggested a reduction in AF recurrence at longer-term follow-up [25], whereas other randomized trials have failed to demonstrate a sustained antiarrhythmic benefit after ablation [26]. Observational data have also shown variable results, with gastrointestinal intolerance being frequently observed and contributing to treatment nonadherence [27].

In our study, colchicine use was not associated with reduced AF or AT recurrence at either 6 or 12 months. Interestingly, although colchicine did not improve clinical rhythm outcomes, patients receiving colchicine demonstrated lower CRP levels at 3 months. This finding suggests a possible anti-inflammatory effect. However, this analysis was exploratory, and the association was no longer significant after adjustment for baseline CRP. Moreover, inflammatory biomarkers were not serially assessed during the 14-day colchicine treatment period; therefore, the 3-month CRP measurement may not adequately reflect the short-term anti-inflammatory effect of colchicine. Accordingly, this observation should be regarded as hypothesis-generating rather than evidence of a causal treatment effect. Multiple factors may explain this neutral finding. First, all patients received peri-procedural corticosteroids shortly after ablation and on the following day. Corticosteroids have been shown to attenuate local atrial inflammatory responses and reduce early arrhythmia recurrence [28,29]. The uniform use of corticosteroids in our cohort may have limited the incremental anti-inflammatory effect attributable to colchicine. Second, colchicine was initiated on the day after the procedure; therefore, its impact on the earliest inflammatory cascade may have been attenuated [19]. Third, because late arrhythmia recurrence is often driven by pulmonary vein reconnection or structural remodeling rather than transient inflammation, short-term anti-inflammatory therapy may have limited influence on long-term rhythm outcomes. Taken together, our findings suggest that adjunctive colchicine therapy did not demonstrate a significant incremental benefit in patients who develop hemopericardium during AF ablation, particularly in the context of routine peri-procedural corticosteroid use. Furthermore, treatment-limiting adverse events occurred in approximately one-third of colchicine-treated patients, highlighting that any potential anti-inflammatory effect should be balanced against treatment tolerability.

### 4.3. Procedural Considerations and Evolving Technologies

The incidence of hemopericardium in our cohort (3.5%) was somewhat higher than that reported in large contemporary registries of AF ablation procedures. Several factors may explain this finding. First, all intraprocedural hemopericardium events requiring pericardiocentesis were prospectively captured, including events occurring during early procedural phases such as transseptal access and electroanatomical mapping. Second, more than half of the patients underwent additional extra-pulmonary vein substrate ablation, reflecting relatively complex procedures with longer procedural duration. Third, the long study period may have contributed to the observed incidence. Finally, differences in case mix and single-center procedural practices compared with multicenter registry populations may also have contributed to the observed incidence [30,31,32]. The study period spanned nine years, during which procedural techniques, lesion strategies, mapping technologies, and operator experience evolved. Because the study was not designed to evaluate temporal trends in complication incidence, the potential contribution of institutional learning-curve effects and temporal procedural changes cannot be fully quantified. Our findings underscore that early detection and prompt drainage are critical determinants of favorable outcomes when this complication occurs. However, because patients without hemopericardium were not included as a comparator group, the present study cannot determine the independent effect of hemopericardium on arrhythmia recurrence. Emerging ablation technologies, including pulsed field ablation (PFA), have been associated with evolving safety profiles in recent registries [33]. Nevertheless, hemopericardium remains a clinically relevant complication in contemporary RFCA practice.

### 4.4. Study Limitations

The present study had several limitations. This was a single-center study, which may limit the generalizability of our findings. Also, only patients who developed hemopericardium during the procedure were included in the analysis; delayed hemopericardium during the hospitalization or after discharge was not included. The precise mechanisms underlying hemopericardium were not systematically adjudicated. Adverse events related to colchicine were not prospectively collected using a standardized surveillance protocol; therefore, adverse events not resulting in treatment discontinuation may have been underreported. The observed differences in age and postprocedural anticoagulation management between groups indicate potential treatment selection bias and confounding by indication. In addition, colchicine administration was non-randomized and determined at the treating operator’s discretion. Furthermore, nearly all patients received peri-procedural intravenous corticosteroids. This concomitant anti-inflammatory treatment may have attenuated post-ablation inflammation and reduced the ability of the present study to isolate any incremental effect attributable to colchicine. The imaging assessment relied solely on transthoracic echocardiography; therefore, detailed evaluation of pericardial inflammatory or fibrotic changes using advanced modalities such as cardiac magnetic resonance or positron emission tomography was not possible. Arrhythmia surveillance was based primarily on scheduled ECGs and 24 h Holter monitoring rather than continuous monitoring. Consequently, asymptomatic or intermittent AF/AT recurrence may have been underestimated compared with strategies using prolonged ECG patches or implantable loop recorders. The multivariable model included several covariates despite a limited number of recurrence events, creating a risk of model overfitting. Accordingly, adjusted estimates should be considered exploratory rather than confirmatory. Finally, the relatively small sample size limits statistical power, particularly for detecting modest differences in arrhythmia recurrence. These findings should be considered hypothesis-generating and warrant confirmation in larger prospective studies.

## 5. Conclusions

In patients with hemopericardium during AF ablation, prompt pericardial drainage is associated with favorable clinical outcomes and minimal long-term sequelae. Adjunctive colchicine therapy was not associated with a significant reduction in arrhythmia recurrence in this setting. However, given the small sample size and limited number of events, a clinically meaningful treatment effect cannot be excluded. Although lower CRP levels were observed at 3 months, this exploratory biomarker finding was not accompanied by improved clinical outcomes. In addition, treatment-limiting adverse events were common, and the present data do not support routine colchicine use after procedural hemopericardium.

## Figures and Tables

**Table 1:** Baseline characteristics of the study population.

	Colchicine	No Colchicine		
	(N = 21)	(N = 54)	*p*-Value	SMD
Age (years)	61.2 ± 10.8	67.4 ± 10.7	0.029	0.570
Male, n (%)	5 (23.8%)	19 (35.2%)	0.501	0.251
Duration of AF (month)	35.3 ± 32.9	23.3 ± 27.4	0.113	0.387
Category of AF, n (%)			0.76	0.200
Paroxysmal	8 (38.1%)	25 (46.3%)		
Persistent	10 (47.6%)	24 (44.4%)		
Long standing persistent	3 (14.3%)	5 (9.3%)		
CHA_2_DS_2_-VASc score	2.0 [1.0; 2.0]	2.0 [1.0; 3.0]	0.126	0.418
Hypertension, n (%)	10 (47.6%)	36 (66.7%)	0.209	0.392
Diabetes mellitus, n (%)	4 (19.0%)	5 (9.3%)	0.256	0.284
Heart failure, n (%)	2 (9.5%)	5 (9.3%)	1	0.009
History of stroke, n (%)	2 (9.5%)	4 (7.4%)	1	0.076
Rhythm at the time of RFCA, n (%)			0.76	0.219
Sinus rhythm	12 (57.1%)	25 (46.3%)		
Atrial fibrillation	8 (38.1%)	26 (48.1%)		
Atrial tachycardia	1 (4.8%)	3 (5.6%)		
LVEF (%)	60.0 [57.0; 64.0]	61.0 [59.0; 64.0]	0.425	0.069
Left atrial diameter (mm)	42.0 [35.8; 44.5]	42.0 [37.0; 46.0]	0.58	0.136
Antiarrhythmic drugs, n (%)			0.75	0.202
Class Ic	17 (81%)	44 (81.5%)		
Class III	4 (19.0%)	9 (16.7%)		
None	0	1 (1.9%)		

Values are mean ± SD, n (%), or median (IQR), SMD: Standardized mean difference, AF; atrial fibrillation, RFCA; radiofrequency catheter ablation, LVEF; left ventricular ejection fraction.

**Table 2:** Procedural characteristics.

	Colchicine	No Colchicine	Total Patients	*p*-Value
	(N = 21)	(N = 54)	(N = 75)	
Hospital stay (days)	4.0 [3.0; 5.0]	5.0 [4.0; 5.0]	5.0 [3.5; 5.0]	0.2
Time from procedure start to PCC (min)	160.0 [136.5; 208.0]	158.5 [120.0; 200.0]	158.5 [124.0; 200.0]	0.88
Total procedure time (min)	200.0 [185.0; 257.0]	223.5 [182.0; 249.0]	219.0 [185.0; 250.0]	0.5
Continuation of RFCA after PCC, n (%)	10 (47.6%)	36 (66.7%)	46 (61.3%)	0.21
Auto transfusion, n (%)	13 (61.9%)	44 (81.5%)	57 (76.0%)	0.14
Amount of auto transfusion (mL)	250.0 [200.0; 300.0]	250.0 [200.0; 450.0]	250.0 [200.0; 450.0]	0.79
Total amount of drainage (mL)	480.0 [320.0; 816.5]	750.0 [370.0; 1000.0]	700.0 [350.0; 950.0]	0.11
ACT level before PCC	343.0 [292.0; 360.5]	350.0 [310.0; 372.0]	348.0 [304.0; 372.0]	0.32
**Laboratory tests**				
Initial Hemoglobin (g/dL)	14.6 ± 1.5	13.4 ± 1.7	13.7 ± 1.7	0.01
Post procedural Hemoglobin (g/dL)	12.9 ± 1.7	11.5 ± 1.7	11.9 ± 1.8	0.003
Change in hemoglobin level (g/dL)	2.3 ± 3.1	1.9 ± 3.2	2.0 ± 3.2	0.64
Initial CRP (mg/dL)	0.05 [0.03; 0.18]	0.06 [0.04; 0.2]	0.06 [0.03; 0.2]	0.38
Postoperative day 1 CRP (mg/dL)	1.7 [0.66; 4.45]	3.7 [1.21; 9.15]	2.8 [1.03; 8.31]	0.14
3-month CRP (mg/dL)	0.03 [0.01; 0.05]	0.05 [0.03; 0.163]	0.05 [0.03; 0.12]	0.02
**Medication**				
Intravenous steroid, n (%)	20 (95.2%)	54 (100.0%)	74 (98.7%)	0.28
NSAID at hospital discharge, n (%)	1 (4.76%)	1 (1.85%)	2 (2.67%)	0.48
Intravenous protamine after RFCA, n (%)	13 (61.9%)	48 (88.89%)	61 (81.3%)	0.7
**Oral anticoagulation**				
Not resuming OAC on the day after RFCA	10 (47.62%)	38 (70.37%)	48 (64.0%)	0.16
Total OAC discontinuation days	0.0 [0.0–1.0]	3.0 [0.0–7.0]	1.0 [0.0; 7.0]	0.002
Taking OAC at hospital discharge, n (%)	19 (90.48%)	34 (62.96%)	53 (70.67%)	0.04

PCC; Pericardiocentesis, NSAID; non-steroidal anti-inflammatory drugs, CRP; C-reactive protein, RFCA; radiofrequency ablation, ACT; activated clotting time, OAC; oral anticoagulants.

**Table 3:** Predictive Factors for 12-month AF/AT recurrence according to Cox Regression Analysis.

	Univariate HR	95% CI	*p*-Value	Multivariate HR	95% CI	*p*-Value
Colchicine	0.96	0.34–2.67	0.93	0.92	0.28–2.98	0.88
Age	1.02	0.98–1.07	0.28	1.02	0.97–1.07	0.49
Sex	0.94	0.36–2.49	0.91	1.03	0.32–3.31	0.96
Prior stroke	1.08	0.24–4.78	0.92	1.16	0.21–6.46	0.86
Paroxysmal AF	0.39	0.14–1.11	0.08	0.22	0.06–0.77	0.02
Duration of AF	1.00	0.99–1.01	0.45	1.00	0.11–0.90	0.30
Hypertension	0.78	0.31–1.95	0.59	0.50	0.17–1.43	0.19
Diabetes mellitus	0.99	0.23–4.32	0.99	0.80	0.11–5.64	0.82
Heart failure	0.73	0.1–5.59	0.77	0.33	0.03–3.3	0.35
LVEF	1.00	0.92–1.09	0.97	1.03	0.92–1.15	0.66
RFCA for extra-PV substrate	0.79	0.32–1.98	0.62	0.43	0.14–1.37	0.15
Continuation of RFCA	0.81	0.32–2.1	0.66	0.82	0.27–2.49	0.72

AF; atrial fibrillation, LVEF; left ventricular ejection fraction, HR; hazard ratio, PV; pulmonary vein.

## Data Availability

The data presented in this study are available from the corresponding author upon reasonable request. The data are not publicly available due to privacy and ethical restrictions related to patient information.

## Supplementary Materials

The following supporting information can be downloaded at: https://www.mdpi.com/article/10.3390/jcdd13090407/s1. Table S1: Atrial structures being ablated at the time of hemopericardium detection and lesions created during the RFCA procedure.

## Abbreviations

The following abbreviations are used in this manuscript:

AF	Atrial fibrillation
AT	atrial tachycardia
ECG	Electrocardiogram
CRP	C-reactive protein
RFCA	Radiofrequency catheter ablation
PV	pulmonary vein
PFA	pulsed field ablation
