# Supplementary material for: Long-Term Clinical Outcomes After Procedural Hemopericardium Requiring Pericardiocentesis During Atrial Fibrillation Ablation
Source: J Cardiovasc Dev Dis. 2026 Aug 24;13(9):407. doi: 10.3390/jcdd13090407 (PMC13607653; doi:10.3390/jcdd13090407)
Supplement: Supplementary file 1 [file jcdd-13-00407-s001.zip › jcdd-4472374-supplementary.pdf]

`

Supplementary Table S1. Atrial structures being ablated at the time of hemopericardium detection and lesions created during the RFCA procedure.

	Colchicine (N=21)	No colchicine (N=54)	Total (N=75)	p-value
**Locations at notification**				0.45
Left side PV	4 (19.0%)	14 (25.9%)	18 (24.0%)	
Rt side PV	6 (28.6%)	8 (14.8%)	14 (18.7%)	
Roof	3 (14.3%)	4 (7.4%)	7 (9.3%)	
Perimitral isthmus	0	6 (11.1%)	6 (8.0%)	
Right atrium	0	4 (7.4%)	4 (5.3%)	
Anterior wall	0	1 (1.9%)	1 (1.3%)	
After procedure	5 (23.8%)	7 (13.0%)	12 (16.0%)	
After EAM map	1 (4.8%)	3 (5.6%)	4 (5.3%)	
Unknown	2 (9.5%)	7 (13.0%)	9 (12.0%)	
**Lesions created**				
PV isolation, n (%)	21 (100.0%)	54 (100.0%)	75 (100.0%)	
Extra-PV, n (%)	14 (66.7%)	29 (53.7%)	43 (57.3%)	0.448
CTI	11 (52.4%)	39 (72.2%)	50 (66.7%)	0.173
Roof	5 (23.8%)	18 (33.3%)	23 (30.7%)	0.6
Marshall bundle	3 (14.3%)	20 (37.0%)	23 (30.7%)	0.101
Inferior line	0	9 (16.7%)	9 (12.0%)	0.05
Perimitral isthmus	7 (33.3%)	22 (40.7%)	29 (38.7%)	0.743
Anterior wall	1 (4.8%)	1 (1.9%)	2 (2.7%)	0.484
SVC	3 (14.3%)	3 (5.6%)	6 (8.0%)	0.34
Any CFAE	2 (9.5%)	1 (1.9%)	3 (4.0%)	0.188

PV; pulmonary vein, CTI; cavotricuspid isthmus, CFAE; complex fractionated atrial electrogram, EAM map; electroanatomic map, SVC; superior vena cava
